# The optimal platelet concentration in platelet-rich plasma for proliferation of human cells in vitro—diversity, biases, and possible basic experimental principles for further research in the field: A review

**DOI:** 10.7717/peerj.10303

**Published:** 2020-11-13

**Authors:** Olav K. Straum

**Affiliations:** Faculty of Humanities, Social Sciences, and Education, UiT The Arctic University of Norway, Tromsø, Norway

**Keywords:** Platelet concentrate, Cell proliferation, Platelet-rich plasma, PRP, Proliferation, Human, In vitro, Growth factor, Platelet lysate

## Abstract

**Background:**

In the last decades, several in vitro studies have tested the effect of plate-rich plasma (PRP) on the proliferation of human cells in search of a wizard for the use of PRP in a clinical setting. However, the literature displays striking differences regarding this question despite the relatively similar experimental design. The aim of this review is twofold: describe and explain this diversity and suggest basic principles for further in vitro studies in the field. The optimal platelet concentration in vivo will also be discussed.

**Methods:**

A search in mainly EMBASE and PubMed was performed to identify in vitro studies that investigate the effect of different PRP concentrations on human cell proliferation. The assessment of bias was based on the principles of “Good Cell Culture Practice” and adapted.

**Results:**

In total, 965 in vitro studies were detected. After the initial screening, 31 studies remained for full-text screening. A total of 16 studies met the criteria of final inclusion and appeared relatively sound. In general, the studies state consistently that PRP stimulates the proliferation of the human cell. Two main types of experimental techniques were detected: 1. *The Fixed PRP Concentration Group* using a fixed PRP concentration throughout the experiment, which leads to a substantial decrease in nutrition available at higher concentrations. 2. *The Fixed PRP Volume Group* using a fixed PRP-to-media ratio (Vol/Vol) throughout the experiment. A general tendency was observed in both groups: when the PRP to media ratio increased (Vol/Vol), the proliferation rate decreased. Further, *The Low Leukocyte group* observed a substantial higher optimal PRP concentration than *The High leukocyte group*. No prominent tendencies was seen regarding anticoagulants, activation methods, and blood donor (age or sex).

**Discussion:**

Two major biases regarding optimal proliferation in vitro is pointed out: *1. Too high PRP volume*. It is speculated that the techniques used by some studies led to an adverse growth condition and even cell starvation at higher concentrations. *2. High leukocyte levels.* Reduced proliferation rate due to proinflammatory substances released during degranulation of leukocytes.

**Conclusions:**

The two main biases may explain the bell-shaped effect of PRP and the detrimental effects at higher platelet concentrations observed in several studies. These biases may also explain the low optimal PRP concentration observed in some studies. Even if one universal optimal PRP concentration does not exist, the review indicates that PRP concentrations in the upper parts of the scale is optimal or at least beneficial. Finally, following basic experimental principles are suggested. 1: The PRP/media ratio (Vol/Vol) should be kept as constant. 2: The PRP/media ratio should provide a sufficient nutrition supply, that is, PRP ≤ 10% (Vol/Vol). 3: The cell density per well (cells/mL) should be defined. 4: Leukocyte level should be kept low, preferable depleted (< 0.1 PLT/µL).

## Introduction

Platelet-rich plasma (PRP) may generally be defined as an autologous concentrate of platelets in a small volume of plasma obtained by centrifugation of venous blood (Engebretsen, Steffen & Alsousou, 2010; Marx, 2004). Originally, PRP was used for hemostasis during surgery and platelet transfusion for patients with thrombocytopenic disorders (Gardener, 1974). However, in the last two decades, the application has expanded to a wide range of medical disciplines, including maxillofacial surgery, dentistry, dermatology, aesthetic surgery, orthopedics, and sports medicine, among others (Anitua, Cugat & Sánchez, 2018; Arshdeep & Kumaran, 2014; Chen et al., 2018; Frautschi et al., 2017; Lemos et al., 2016).

The biological rationale for the use of PRP is the wound healing and regenerative properties of the platelets. Platelets hold about 50–80 *α*-granules that contain hundreds of bioactive proteins, including a wide range of growth factors (Blair & Flaumenhaft, 2009; Neumüller, Ellinger & Wagner, 2015). The most important growth factors in this context are platelet-derived growth factor (PDGF), vascular endothelial growth factor (VEGF), fibroblast growth factor (FGF), transforming growth factor-beta 1 (TGF-β1), epidermal growth factor (EGF), insulin-like growth factor (IGF), connecting tissue growth factor (CTGF), and hepatocyte growth factor (HGF) (Dhurat & Sukesh, 2014)

During platelet activation, the *α*-granules are fused with the membrane of the open canalicular system (OCS) inside the platelet (Blair & Flaumenhaft, 2009). Here, the growth factors are processed to the bioactive form before exocytosed through the platelet membrane. In connection with and parallel to exocytosis, the platelet undergoes dramatic morphological changes seen in the microscope as increased hyalomere and a centralized and constricted granulomere, which give the activated platelets the characteristic pseudopodic/filopodic shape (Twomey et al., 2018). The active growth factors are secreted and bind to the transmembrane receptors in the cells in the actual tissue area (Antoniades & Williams, 1983; Schliephake, 2002). This stimulates cell growth, mitogenesis, and chemotaxis, which leads to proliferation and extracellular matrix formation, and thus tissue repair and tissue regeneration. Cells in the mesenchymal linage like osteoblasts, fibroblasts, endothelial cells, and epidermal cells are particularly responsive to these growth factors (Blair & Flaumenhaft, 2009; Van der Heiden, Cantley & Thompson, 2009). The whole process requires a dramatic increase in cellular nutrition uptake (Thompson & Bielska, 2019; Van der Heiden, Cantley & Thompson, 2009). The basic idea of PRP treatment is to enhance this natural regeneration process through a concentrated dosage of platelets and increased levels of growth factors compared to peripheral blood levels.

During activation, platelets also release platelet-derived microparticle (PDM) (Neumüller, Ellinger & Wagner, 2015). These membrane generated vesicles, which range from 0.1–1 µm in diameter, may play a role regarding the generative effect of PRP. Research indicates that PDM may “stimulate the release of cytokines, activate intracellular signaling pathways, promote angiogenesis, and are involved in tissue regeneration and cancer metastasis” (Varon et al., 2012). However, this aspect has not been thematized in the included studies and will not be investigated further.

### The aim

In the last two decades, several in vitro studies have been performed to study the regenerative effect of PRP on different types of cells in culture and to establish an optimal platelet concentration in PRP for tissue regeneration. Cell proliferation has been the focus, but other important aspects in the regenerative process have also been investigated, such as the effect of PRP on cell migration, gene expression, and exocytosis of extracellular substances, for example, collagen type I and III, and glycosaminoglycan, among others. However, the literature displays striking differences regarding the most effective platelet concentration for proliferation, and other regenerative aspects despite their relatively similar experimental design. This has led to different clinical recommendations and treatment regimes. Some consider a high or very high concentration as most beneficial ( Jo et al., 2012), while others advocate low or moderate platelet concentrations and consider very high concentrations of PRP to be counterproductive with a potential risk of cell death (Giusti et al., 2014, Kakudo et al., 2008, Zhou et al., 2016). Both positions refer to different in vitro studies, and the question of which perspective on PRP concentration is valid is still open (Smith et al., 2019). Also the PRP production methods and thus the biological and biochemical characteristic of PRP is varying. Several reviews have incorporated the question regarding the optimal platelet concentration, but not in a comprehensive way (Liu et al., 2008; Setayesh et al., 2018; Smith et al., 2019).

Therefore, the aim of this review is twofold:

1. Clarify and explain the inconsistency among in vitro studies regarding the optimal platelet concentration for proliferation of human cells
2. Suggest basic principles for further in vitro studies

The author will also discuss possible implications for the most effective PRP concentration in vivo.

### Clarification of concepts

Although the studies often apply the term “PRP concentration” or “platelet concentration,” PRP was infrequently added directly to the culture wells. After the PRP was processed, almost all research groups activated, incubated, and centrifuged the PRP to obtain a supernatant rich in growth factors. This supernatant has been given different names in the literature, for example, “PRP-releasate,” “supernatant rich in growth factor,” “platelet lysate,” “preparation rich in growth factors,” “platelet-rich clot releasate,” among others. In this review, “PRP-lysate” will be used in the author’s discussion. The next step in the experiment was to add the PRP-lysate to the cell culture at various concentrations corresponding to a given platelet concentration. Thus, the terms “PRP concentration” and PRP-lysates are used interchangeably in the studies and this review.

## Review Methodology

### Search strategy

Initially, a comprehensive search was carried out in EMBASE OvidSP and PubMed. The search terms used included: “platelet-rich plasma” OR “platelet concentrate*” OR “thrombocyte concentrate*” AND proliferation AND “in vitro”. Relevant articles found in references were also added. The search was performed in January 2020 and repeated in March 2020. The search was limited to peer reviewed literature in English. When the duplicates in and across the two databases were removed, the records were screened in accordance with the criteria of inclusion and exclusion.

### Criteria of inclusion

The minimum criteria for inclusion were controlled experimental studies testing the effect of different PRP concentrations on cell proliferation. Nevertheless, several studies also included other important regenerative parameters such as cell motility and synthesis of important extracellular substances. These findings are also presented in the review but are not a main focus. The number of in vitro studies utilizing human cells has grown substantially in the last two decades. Therefore, only articles based on human cells were included. Since the effect of different concentrations of platelets is the main focus, only studies that utilized three different PRP concentrations or more were included.

### Criteria of exclusion

A large number studies tested the proliferation effect of PRP in combination or as a comparison to different types of biomaterials. To achieve a minimum of homogeneity with respect to experimental design, these studies were excluded. Studies that utilized a low maximum PRP concentration, here defined as a concentration range lower than threefold of the baseline of whole blood (WB) or approx. 600 million plt/µL as maximum, were excluded, for example, De Mos, van der Windt & Jahr, (2008), Atashi et al., (2015), and partly Cavallo et al., (2014). To be able to make comparisons between the studies, articles that did not disclose platelet concentrations in plt/µL or fold, but focused solely on the levels of growth factors (pg/mL) were excluded, for example, Han et al. (2007). The actual platelet count is also important for the PRP processing in an in vivo setting. Even if cells in the mesenchymal line are prioritized, adipocytes were excluded due to a musculoskeletal focus. However, studies that utilized HUVECs were included since angiogenesis, in general, is an important aspect of tissue regeneration and because HUVECs often are used as a laboratory model system for the study of angiogenesis. Some studies included in addition cell types not directly relevant in a musculoskeletal context. These cell types were included but not emphasized. Finally, editorials and letters to the editors were also excluded. A schematic presentation of the search strategy is presented in the PRISMA 2009 Flow Diagram (Appendix S1).

The included studies will be tabulated and discussed chronologically (by publishing year) and alphabetically in each year group. A condensed overview of this article is presented in the PRISMA 2009 Checklist (Appendix S2).

### The assessment of risk of biases

For in vitro studies, no standardized international tool of bias assessment exists, although candidates are developing (Hartung et al., 2019). The author applied as a starting point the principles of Good Cell Culture Practice (GCCP) (Hartung et al., 2019) and further developed these principles to the actual field. The principles regarding cell description, culture media content, and method/reproducibility were especially emphasized. The following basic parameters were selected as potential areas of biases.

1. Cell type(s)
2. Cell site origin
3. Media description
4. Sample size (number of blood donors)
5. Number of wells used for testing of each PRP concentration
6. Duration of PRP exposure
7. Materials used according to the manufacturers’ instructions
8. Cell number per well
9. PRP-to-media ratio

If a study reported or did not report a parameter, a “Yes” or a “No” was noted, respectively. The classification was as follow.

• Studies that reported up to 5 parameters were also classified as “high risk of biases.”
• Studies that reported 6–7 parameters were classified as “moderate risk of biases.”
• Studies that reported 8–9 parameters were classified as “low risk of biases.”

## Results

### Study selection

In total, 965 records were identified: 426 studies in EMBASE OvidSP, 539 in Pubmed. The duplicates in the two bases were excluded; the initial number of studies was 525. After the first screening (title and abstract), 495 studies were excluded, frequently due to the use of animal cells, no focus on different PRP concentration, or because the design combined PRP with different types of biomaterials, etc. An additional records were identified in references. The remaining 31 papers were included for full-text screening, of which 16 papers were included for the final analysis.

### Risk of bias

In general, the 16 studies appeared relatively sound. Based on the basic bias assessment criteria mentioned above, no studies were classified as “high risk of biases,” and only 4 studies were classified as “moderate risk of biases” (Haynesworth et al., 2002; Mishra et al., 2009; Wang et al., 2012; Sadoghi et al., 2013). The remaining 12 studies were classified as “low risk of biases.” The fifth parameter (Number of wells tested for each PRP concentration) was the weakest point with a total of 8 negatives (Table 1).

**Table 1:** Risk of bias assessment.

**Study**	**Cell type(s) tested**	**Cell siteorigin**	**Media description**	**Number ofblood donors**	**Number of wells tested for each PRP concentration**	**Duration of PRP exposure**	**Materials used according to manufacturers’instructions**	**Cell numberper well or cm**^**2**^	**PRP/lysate to media ratio**	**Risk of bias**
Haynesworth et al. (2002)	Yes	Yes	Yes	**No**	**No**	Yes	Yes	Yes	Yes	***Moderate***
Graziani et al. (2006)	Yes	Yes	Yes	Yes 3	Yes	Yes	Yes	Yes	Yes	**Low**
Rughetti et al. (2008)	Yes	Yes	Yes	Yes 8	Yes 3	Yes	Yes	Yes	**No**	**Low**
Anitua et al. (2009)	Yes	Yes	Yes	Yes 2	**No**	Yes	Yes	Yes	Yes	**Low**
Hsu, Kuo & Tseng (2009)	Yes	Yes	Yes	Yes 20	Yes	Yes	Yes	Yes	Yes	**Low**
Mishra et al. (2009)	Yes	**No**	Yes	**No**	**No**	Yes	Yes	Yes	Yes	***Moderate***
Chen et al. (2012)	Yes	Yes	Yes	Yes 10	Yes	Yes	Yes	Yes	Yes	**Low**
Jo et al. (2012)	Yes	Yes	Yes	Yes 9	Yes	Yes	Yes	Yes	Yes	**Low**
Mazzocca et al. (2012)	Yes	Yes	Yes	Yes 8	Yes	Yes	Yes	Yes	Yes	**Low**
Wang et al. (2012)	Yes	**No**	Yes	Yes 3	**No**	Yes	Yes	Yes	Yes	***Moderate***
Sadoghi et al. (2013)	Yes	Yes	Yes	**No**	Yes	Yes	Yes	Yes	**No**	***Moderate***
Amable et al. (2014)	Yes	Yes	Yes	Yes 3 pr. pool	**No**	Yes	Yes	Yes	Yes	**Low**
Giusti et al. (2014)	Yes	Yes	Yes	Yes 3	Yes	Yes	Yes	Yes	**No**	**Low**
Tavassoli-Hojjati et al. (2016)	Yes	Yes	Yes	Yes 1	**No**	Yes	Yes	Yes	Yes	**Low**
Berger et al. (2019)	Yes	Yes	Yes	Yes 14	**No**	Yes	Yes	Yes	Yes	**Low**
Wang et al. (2019)	Yes	Yes	Yes	Yes 8	**No**	Yes	Yes	Yes	Yes	**Low**

### The effects of PRP in vitro: an overview

In general, all the studies consistently report that PRP may increase cell proliferation in vitro (Table 2). An exception is skin fibroblasts in the study of Anitua et al. (2009). Different studies assayed different extracellular substances and gene expression, and thus difficult to compare systematically. Still, three studies observed an increased synthesis of collagen type I and/or II or HA (Anitua et al., 2009; Jo et al., 2012; Wang et al., 2012). Four studies observed increased cell motility and invasion at moderate (Berger et al., 2019; Giusti et al., 2014; Graziani et al., 2006) or relatively high concentration (Rughetti et al., 2008).

**Table 2:** Descriptive overview.

**Study**	**PRP concentrations tested(plt/**μ**L or fold of WB)**	**Cell type tested**	**Significant stimulation** of cell proliferation	**Optimal PRP concentrationcell proliferation(Vol/Vol and plt/**μ**Lor fold)**	**Optimal PRP concentration cell motility andInvasion (plt/**μ**L)**	**Exocytation and extracellular matrix**
Haynesworth et al. (2002)	0.625-fold, 1.25-fold, 2.5-fold and 5-fold	HMSC	Yes	**10% of 1.6 × 10**^**6**^**(5 folds over baseline)**	Not tested	Not tested
		Fibroblasts	Yes			
Graziani et al. (2006)	2.5-fold, 3.5-fold and 4.2–5.5-fold (PRP max)	Osteoblasts	Yes	**33.3% of 2.5 × ≈ 0.570 × 10**^**6**^	33.3% of 2.5 × Ca 0.570 ×10^6^	OPG upregulated at 2.5x. OCN and TGF- *β*1 upregulated at 4.2-5.5x (PRP-max)
		Fibroblasts	Yes	**33.3% of2.5 × ≈ 0.570 × 10**^**6**^		
Rughetti et al. (2008)	0.3 ×10^6^, 0.5 × 10^6,^ 0.75 ×10^6^, 1,25 ×10^6^, 1.75 ×10^6^, 2.25 ×10^6^, 2.75 ×10^6^, 3.25 ×10^6^, 4. × 10^6^, 5 ×10^6^ and 7 ×10^6^	Endothelial cells (umbilical vein)	Yes	**1.25 × 10**^**6**^**(PRP/media ratio unclear)**	1.5 ×10^6^ (PRP/media ratio unclear)	Not tested
Anitua et al. (2009)	0.16 ±1 ×10^6^ 0.404 ±39 ×10^6^ and 0.767 ±95 ×10^6^/ μ L (2x and 4x of baseline) PRP to media ratio of 20%/80%	Fibroblasts (skin)	No	**________**	Not tested	Significant increase in collagen I and HA
		Fibroblasts (synovium)	Yes	**20% of 0.767 ± 95 × 10**^**6**^**and0.404 ± 39 × 10**^**6**^		
		Fibroblasts (tendon)	Yes	**20% of 0.767 ± 95 × 10**^**6**^**and 0.404 ± 39 × 10**^**6**^		
Hsu, Kuo & Tseng (2009)	1.124 ×10^6^ plt/μ L was added to the wells in concentrations of 2%, 5%, 15%, and 30%	Fibroblasts (PDL)	Yes	**5% of 1.124 × 10**^**6**^**of5% (Vol/Vol)**	Not tested	(Angiogenesis Inhibitor in PRP)
		Osteoblasts				
		HUVEC				
Mishra et al. (2009)	1 ×10^6^/ μ L(non-activated) added to media at ratios of 1%, 5%, 10% and 20% (Vol/Vol) For HMSC only 10% PRP	Fibroblasts (skin)	Yes	**10% of 1 × 10**^**6**^	Not tested	Osteogenic marker RUNx2 doubled. Chondrogenic marker Sox-9 mRNA increased tenfold (HMSC)
		HMSC	Yes			
Chen et al. (2012)	Lysate from PRP of 1.0 × 10^6^ added to media at ratios of 0%, 1%, 5%, 10% lysate (Vol/Vol)	Dental pulp stem cells	Yes	**5% of 1.0 × 10**^**6**^	Not tested	(Cell differentiation)
Jo et al. (2012)	0.1, 0.2, 0.4, 0.8, 1, 2, 4, 8 and 16 ×10^6^	Tenocytes (rotary cuff)	Yes	**10% of 4.00 × 10**^**6**^**(CaCl**_**2**_**)and10% of 8.00 × 10**^**6**^**(CaCl**_**2**_**+ thrombin)**	Not tested	Significant increase in collagen I and III and glycosamino-glycan
Mazzocca et al. (2012)	PRP_LP_: 382.0+/-111.6 ×10^3^/ μ L PRP_DS_: 472.6+/-224.2 ×10^3^/ μ L PRP_HP_: 940.1+/-425.8 ×10^3^	Myocytes	Yes	**10% of PRP**_**L**__**P**_**: 382.0+/-111.6 × 10**^**3**^	Not tested	Significantly increased growth factors in all three PRPs. Highest PRP_HP_(940.1+/- 425.8 ×10^3^/ μ L)
		Osteoblasts	Yes	**10% ofPRP**_**DS**_**: 472.6+/-224.2 × 10**^**3**^		
		Tenocytes	Yes	**No significance btw PRP**_**LP**_**, PRP**_**DS,**_**and PRP**_**HP**_		
Wang et al. (2012)	1.2–1.9 ×10^6^/ μ L 1%, 5% and 10% (Vol/Vol)	Tenocytes	Yes	**10% of 1.5-1.9 × 10**^**6**^**(4x)**	Not tested	Significant increased collagen syntesis in 5-1.9 ×10^6^/ μ L (4x) at 10% PRP2
Sadoghi et al. (2013)	1-, 5-, and 10-fold PRP was obtained by diluting initial PRP in PPP. The PRP/media ratio is unclear.	Fibroblasts (rotary cuff)	Yes	**5-fold ≈ 1.25 × 10**^**6**^**(PRP/media ratio unclear)**	Not tested	(Cell differentiation)
Amable et al. (2014)	2.94+/-1.9 ×10^6^ plt/μ L was mixed with culture media ( *α*-MEM) to obtain following PRP concentrations: 1%, 2.5%, 5%, 10%, 20%, 30%, 40% and 50% (Vol/Vol)	HMSC (bone marrow)	Yes	**10% of 2.94+/-1.9 × 10**^**6**^	Not tested	Various reaction to PRP depending on cell type
		HMSC (adipose tissue)				
		HMSC (Wharton‘s Jelly)				
Giusti et al. (2014)	0.5 ×10^6^, 1 ×10^6^, 2 ×10^6^, 3 × 10^6^ and 5 ×10^6^/ μ L	Tenocytes	Yes	**0.5 × 10**^**6**^**(PRP/media ratio unclear)**	0.5 ×10^6^ at 46 h (PRP/media ratio unclear)	Significant dose-dependent increase in MMP up to 5 ×10^6^/ μ L and collagen I at 1 ×10^6^ and2 ×10^6^/ μ L
Tavassoli-Hojjati et al. (2016)	1.194 ×10^6^/ μ L diluted in DMEM resulting in concentration of 0.1%, 5% and 50%.	Fibroblasts (periodontal ligament)	Yes	**5% of 1.194 × 10**^**6**^	Not tested	Not tested
Berger et al. (2019)	Platelet lysate corresponding to platelet levels of 14x, 7x, 3.5x, 1.75x and 0.9x of WB	Fibroblasts (Achilles, patellar, and palmaris)	Yes	**20% of0.875 × 10**^**6**^**(Young group)**	______ **Old group:3.5 × 10**^**6**^	Not tested
				**20% of3.5 × 10**^**6**^**(Old group)**		
Wang et al. (2019)	Platelet lysate corresponding to 0.2 ×10^6^, 0.5 ×10^6^, 0.8 ×10^6^, 1.0 ×10^6^, 1.2 ×10^6^, 1.5 ×10^6^, 2.0 ×10^6^, 2.7 ×10^6^, and 3.0 ×10^6^	HMSC (bone marrow)	Yes	**10% of 1.5 to 3.0 × 10**^**6**^	Not tested	(Cell differentiation)

### Two main types of experimental technique

This review detected two main types of experimental techniques:

*1. The Fixed PRP Concentration Group*: In six of the studies, a fixed initial PRP concentration was used. Different concentrations were achieved by varying the PRP-to-media ratio (Vol/Vol) in which the cells were cultured. Mishra et al. (2009) and Wang et al. (2019) had similar initial PRP concentration (1.0 and 1.55 ×10^6^ plt/µL, respectively). They found an increase in proliferation up to 10% PRP and a decrease when a volume of 20% PRP was used. Amable et al. (2014) also observed an increase in cell proliferation up to 10% PRP and a decline when higher volume concentrations were used. The fixed PRP concentration was 2.94 ± -1.9 ×10^6^plt/µL. Hsu, Kuo & Tseng (2009), Chen et al. (2018) and Tavassoli-Hojjati et al. (2016) used an initial PRP concentration of 1.124, 1.0 ×10^6^ and 1.194 ×10^6^, respectively, and observed a maximal proliferation at 5% PRP. However, in Hsu, Kuo & Tseng (2009) and Tavassoli-Hojjati et al. (2016), a volume of 10% was not a part of the coarse test scale, and when the next concentration was tested (15% and 50% PRP), the proliferation rate declined. Giusti et al. (2014) seem to belong to this group, but do not clarify the PRP/media ratio (Vol/Vol) and thus difficult to compare to the others (Valerio et al., 2012). It is difficult to compare the studies in an accurate metric manner due to different initial PRP concentrations and different PRP volume concentrations tested. Despite heterogeneity, this group loosely indicate that PRP of 5–10% (Vol/Vol) at a platelet concentration of 1–1.5 ×10^6^ is the most effective in vitro. In general, the technique of this group leads to a substantial decrease in nutrition available at higher concentrations: when the PRP concentration (volume) increases, the culture medium (nutrition) will decrease (Table 3). In the Discussion, I will argue that this experimental technique probably is encumbered with biases regarding the optimal PRP concentration in vivo.

**Table 3:** The Fixed PRP Concentration Group.

**Study**	**Diluting procedure**	**Fixed PRP concentration proliferation(plt/**μ**L)**	**Optimal PRP/media ratio(Vol/Vol) for cell proliferation**
Hsu, Kuo & Tseng (2009)	PRP of 1.124 ×10^6^ plt/μ L was added to the wells in concentrations of 2%, 5%, 15%, and 30%	1.124 ×10^6^	5%
Mishra et al. (2009)	A standardized PRP containing 1 million plt/ μ L was added to the culture at a ratio of 0.1%, 1%, 5%, 10% and 20% (Vol/Vol).	1.0 ×10^6^	10%
Chen et al. (2012)	PRP of 1.0 ×10^6^ plt/μ L was added to media (*α*-MEM) at ratios of 0%, 1%, 5%, 10% (Vol/Vol)	1.0 ×10^6^	5%
Wang et al. (2012)	1.25, 1.5 and 1.9 ×10^6^ (mean 1.55 ×10^6^) was added to the culture at a ratio of 1%, 5% and 10% to media (Vol/Vol)	1.55 ×10^6^	10%
Tavassoli-Hojjati et al. (2016)	Initial PRP contained an average platelet count of 1,194,000/ μ L. The concentrations of 0.1%, 5%, and 50% was obtained by diluting initial PRP in DMEM.	1,194 ×10^6^	5%
Amable et al. (2014)	PRP of 2.94+/-1.9 ×10^6^ plt/μ L was mixed with culture media ( *α*-MEM) to obtain following PRP concentrations: 1%, 2.5%, 5%, 10%, 20%, 30%, 40% and 50% (Vol/Vol)	2.94+/-1.9 ×10^6^	10%
Giusti et al. (2014)	Initial PRP contained 4.5 ×10^6^ to 6 ×10^6^ plt/ μ L. The cells were treated with PRP-lysate which was diluted in culture medium + 1% FDS to obtain 0.5 ×10^6^, 1 ×10^6^, 2 ×10^6^, 3 ×10^6^, and 5 ×10^6^ plt/ μ L.	Appr. 4.5 ×10^6^ to 6 ×10^6^	Not described

*2. The Fixed PRP Volume Group*: The other studies utilized a fixed ratio of PRP to culture media (Vol/Vol) throughout the experiment, for example, 10%/90% or 20%/80%. Different PRP concentrations were achieved by diluting the initial PRP-lysate within the chosen fixed volume of PRP, frequently in MDEM or *α*-MEM. Variation in PRP-to-media ratio (Vol/Vol) was limited to the fixed PRP volume. Haynesworth et al. (2002), Jo et al. (2012), and Wang et al. (2019) used a ratio of 10% PRP (Vol/Vol) and observed an optimal proliferation rate at 1.6 ×10^6^, 4.0 ×10^6^ (fibroblast), and 1.5 ×10^6^ plt/µL, respectively. Similar results found by Rughetti et al. (2008). Anitua et al. (2009) and Berger et al. (2019), on the other hand, used a PRP volume of 20% and found maximum proliferation rate at 0.767 ± .95 and 0.875 (young group) x 10^6^ plt/µL, respectively—about half of Haynesworth et al. (2002) and Wang et al. (2019). Graziani et al. (2006) used the highest PRP/media ratio (33%/67%) and observed maximum proliferation of approx. 0.570 ×10^6^ (osteoblast) and 0.228 ×10^6^ (fibroblast) plt/µL (Tavassoli-Hojjati et al., 2016). Unfortunately, Rughetti et al. (2008) and Sadoghi et al. (2013) did not clarify the exact ratio, which prevents a full comparison in the group. Sadoghi et al. found the optimal platelet concentration to be fivefold, and if we assume an average baseline in WB of 0.25 ×10^6^ plt/µL, the maximum proliferation can be estimated to be 1.25 ×10^6^ plt/µL [45]. When Haynesworth et al. (2002) increased the PRP-to-media ratio to 20%/80% (Vol/Vol) to obtain a platelet concentration of 10-fold (3.2 ×10^6^ plt/µL), they observed a decrease in the proliferation rate (Hartung et al., 2019). One trait that emerges in this group is that studies using a fixed volume of PRP higher than 10% (Vol/Vol) observed lower optimal platelet concentration for proliferation. Still, Mazzocca et al. (2012) differs from this trait and found that relatively moderate platelet concentrations as most effective, even if PRP/media ratio of 10%/90% was used (Table 4).

**Table 4:** The Fixed PRP Volume Group.

**Study**	**Diluting procedure**	**Fixed PRP/media ratio (Vol/Vol)**	**Optimal concentration forproliferation (plt/**μ**L)**
Haynesworth et al. (2002)	The initial PRP contained 1,600 ×10^3^ plt/μ L (5fold). PRP-lysates corresponding to PRP concentrations of 0.625, 1.25, and 2.5 was made by diluting the lysate in MDEM. Each concentration was added to the media in a ratio of 10%/90% (Vol/Vol)	10%	1.6 × 10^6^
Rughetti et al. (2008)	Initial PRP was activated and diluted in DMEM + 2.5% FCS (proliferation) or in MDEM only (motility and invasion). Platelet concentration at 3 ×10^5^, 5 ×10^5^, 7.5 ×10^5^, 1.25 ×10^6^, 1.75 ×10^6^, 2.25 ×10^6^, 2.75 ×10^6^, 3.25 ×10^6^, 4 ×10^6^, 5 × 10^6,^ and 7 × 10^6^plt/ μ L was added to the medium	Not described Probably a constant ratio	1.25 ×10^6^
Jo et al. (2012)	10% activated PRP was added to the culture media (Vol/Vol) at platelet concentrations of 100, 200, 400, 800, 1000, 2000, 4000, 8000 and 16,000 ×10^3^/ μ L.	10%	4.0 × 10^6^(CaC_2_)
			8.0 ×10^6^ (CaC_2_ + trombine)
Sadoghi et al. (2013)	1-, 5-, and 10-fold PRP was obtained by diluting initial PRP in PPP. The PRP/media ratio is unclear.	Not described	5-fold
Anitua et al. (2009)	200% and 400% of WB baseline Appr. platelet concentration of 404 ±39 × 10^3^ and 767 ±95 × 10^3^ added to media in a 20%/80% ratio	20%	0.767 ± .95 × 10^6^
			No sign. difference between PRP and controls regarding skin fibroblasts
Graziani et al. (2006)	Initial PRP contained 800,000-1,37,00 plt/ μ L. Maximum PRP-lysate (PRP-max) was diluted in DMEM to obtain PRP-lysate containing 250%, 350% over WB baseline	33% (100ul culture media and 50ul PRP-lysate of any concentration)	2,5x ca.0.570 ×10^6^ (osteoblast) 1x ca. 0.228 ×10^6^ (fibroblast)
Mazzocca et al. (2012)	PRP_LP_ (382.0+/-111.6 × 10^3^plt/ μ L) PRP_DS_ (472.6+/-224.2 ×10^3^ plt/ μ L) PRP_HP_ (940.1+/-425.8 ×10^3^ plt/ μ L)	10%	382.0+/-111.6 × 10^3^ (PRP_LP_) - Myocytes
			472.6+/-224.2 × 10^3^ ( PRP_DS_)- Osteoblasts
			382.0+/-111.6 × 10^3^ (PRP_LP_) -Tenocytes
Berger et al. (2019)	Platelet lysate (PL) was diluted in PPP to obtain lysates corresponding to platelet levels of 14x, 7x, 3.5x, 1.75x and 0.9x of WB.	20%	Young group: 0.875 ×10^6^
			Old group: 3.5 ×10^6^
Wang et al. (2019)	Platelet lysate corresponding to 0.2 ×10^6^, 0.5 ×10^6^, 0.8 ×10^6^, 1.0 ×10^6^, 1.2 ×10^6^, 1.5 ×10^6^, 2.0 ×10^6^, 2.7 ×10^6^, and 3.0 ×10^6^	10%	1.5–3.0 ×10^6^

### Cell type and cell site origin

This review indicates that the effect of PRP is varying according to cell type. According to Mishra et al. (2009), HMSC was more responsive to PRP than fibroblasts. Within the frame of the same experimental design, Graziani et al. (2006) observed that osteoblast was more receptive to a higher PRP concentration than fibroblasts. According to Jo et al. (2012), the maximal proliferation rate for fibroblasts and HMSC was 4 ×10^6^ and 8 ×10^6^, respectively.

The cell site origin might also be a factor. Anitua et al. (2009) showed that fibroblasts from the skin did respond equally to PRP and PPP, while PRP significantly stimulated the proliferation of fibroblasts from synovium and tendon in a dose-dependent manner. Regarding tendonal fibroblasts, earlier studies also report that tendons from different sites have different tissue structures, composition, cell phenotypes, and metabolic characteristics. Further research is needed to elucidate how different PRP concentrations affect different types of cells and how the same type of cells harvested from different sites respond.

### PRP preparation method and biological and biochemical characteristics of PRP

This review clearly states the pronounced heterogeneity regarding PRP preparation methods. The differences apply to spinning technique, use of anticoagulant, PRP activation method, and thus biological properties of the final PRP product.

#### Spinning techniques

Regarding the spinning technique, that is, g-force, spinning time, and, in some extent, temperature, the diversity makes it almost impossible to compare the studies. In some of the studies, an ordinary table centrifuge is utilized; others used advanced plateletpheresis system, and others a commercial centrifuges specialized for PRP production. More important, five of the studies did not clarify the g-force utilized during the process or inform only about the RPM (Hsu, Kuo & Tseng, 2009; Mazzocca et al., 2012; Rughetti et al., 2008; Tavassoli-Hojjati et al., 2016; Wang et al., 2019). Others used a commercial PRP centrifuges without clarifying the spinning parameters (Haynesworth et al., 2002; Mishra et al., 2009).

#### Leukocyte levels and biochemical components in PRP

The leukocyte levels in PRP are of particular importance, especially in vitro, due to possible host-donor reactions. The reviewed studies can roughly be divided into two groups:

• *The High Leukocyte Group*—five studies applied PRP with an increased leukocyte level compared to WB, often incorporating the buffy coat in the PRP. The PRP product in this group may be characterized as L-PRP (Table 5)
• *The Low Leukocyte Group*—seven studies applied PRP with a decreased leukocyte level compared to WB, often using a leukocyte filter or carefully avoiding the buffy coat. The PRP product in this group may be characterized as P-PRP (Table 6)

Rughetti et al. (2008), Hsu, Kuo & Tseng (2009), and Chen et al. (2012) are excluded in this comparison because the author is not able to determine the leukocyte characteristics. Mazzocca et al. (2012) is also excluded due to the large deviation regarding initial PRP concentrations. As Tables 5 and 6 demonstrate, several studies did not clarify the leukocyte level in a metrical manner, and the author had to interpret the described PRP protocol to determine whether the level was decreased or increased compared to WB baseline. A crucial point in the interpretation is whether the buffy coat was included after the first spin or not and/or if the leukocytes were removed during the second or eventually third spin. The studies that utilized and referred to a specific brand of commercial PRP equipment are a challenge, and the appraisal was based on the manufacturer’s description.

**Table 5:** The High Leukocyte Group.

**Study**	**Leukocyte characteristic**		**Anti-coagulant**	**Activation method**	**Optimal PRP concentration(Vol/Vol and plt/ μ L or fold)**
Haynesworth et al. (2002)	**Exact leukocyte levels not clarified.** Symphony™ Platelet Concentration System (DePuy AcroMed, Raynham, MA)	**High(?)**	**ACD-A**	**Thrombine+CaCl**_**2**_	**10% of 1.6 × 10**^**6**^**(5 folds over baseline)**
Mishra et al. (2009)	**Exact leukocyte levels not clarified.** Medtronic Magellan device (Medtronic, Minneapolis MN)	**High(?)**	**ACD**	**No activation**	**10% of 1 × 10**^**6**^
Sadoghi et al. (2013)	**Exact leukocyte levels not clarified.** “While erythrocytes were discarded, the blood plasma and a buffy coat of PRP were harvested to prepare PRP”.	**High**	**Sodium citrate**	**Thrombine+CaCl**_**2**_	**5-fold ≈ 1.25 × 10**^**6**^**(PRP/media ratio unclear)**
Giusti et al. (2014)	**Exact leukocyte: 1. 17,010, 2. 8100 and 24,000 WBC/μ L** (Table 1 in the article)	**High**	**GPD**	**Thrombin+Calcium gluconate**	**0.5 × 10**^**6**^**(PRP/media ratio unclear)**
Tavassoli-Hojjati et al. (2016)	**Exact leukocyte levels not clarified.** “The whole blood was initially centrifuged at 2,400 rpm for 10 min to separate red blood cell (RBC) portion from PRP and platelet-poor plasma. The upper layer of RBC fraction and PRP portion were removed and centrifuged again at 3,600 rpm for 15 min, and PRP was extracted in a plain collection tube (BD, United States)”.	**High**	**ACD-A**	**CaCl**_**2**_	**5% of 1.194 × 10**^**6**^
**Mean optial PRP concentration**					**0.81 × 10**^**6**^

**Table 6:** The Low Leukocyte Group.

**Study**	**Leucocyte characteristic**		**Anti-coagulant**	**Activationmethode**	**Optimal PRP concentration(Vol/Vol and plt/ μ L or fold)**	**Number used for calculation**
Graziani et al. (2006)	**Exact leukocyte levels not clarified.** “The platelets were automatically leukodepleted by negative charged pall filter.”	**Low**	**ACD-A**	**Autologous throm.+Ca. gluc.**	**33.3% of 2.5x ≈ 0.570 × 10**^**6**^	**0.570 × 10**^**6**^
Anitua et al. (2009)	**Leukocyte level:<200 PLT/μ L** (see Table 1 in their article) “care was taken to avoid the buffy coat.”	**Low**	**Sodium citrat**	**CaCl**_**2**_	**20% of 0.767 ± 95 × 10**^**6**^**and0.404 ± 39 × 10**^**6**^	**0.767 ± 95 × 10**^**6**^
Jo et al. (2012)	**Leukocyte level:** Mean RBC and WBC counts reduced from 4.48 ± 0.31 and 6.11 ± 1.56 in whole blood to 0.15 ± 0.06 and 0.01 ± 0.01 in PRP, respectively (*P* < 001).	**Low**	**ACD**	**Ca. gluc. orThrom.+ Ca. gluc.**	**10% of 4.00 × 10**^**6**^**(Ca. gluc.)and10% of 8.00 × 10**^**6**^**(Ca.gluc. + throm.)**	**4.00 × 10**^**6**^
						**8.00 × 10**^**6**^
Wang et al. (2012)	**Exact leukocyte levels not clarified.** “Briefly, whole blood was centrifuged at 300g for 10 min and the generated blood monocyte layer was further centrifuged for 10 min and the supernatant was collected.”	**Low (?)**	**Sodium citrate**	**CaCl**_**2**_	**10% of 1.5-1.9 × 10**^**6**^**(4x)**	**1.7 × 10**^**6**^
Amable et al. (2014)	**Exact leukocyte levels not clarified.** “Briefly, blood harvested in ACD-containing tubes (BD, #364606) was centrifuged during 5 min at 300 g. After separating the platelet-containing plasma above the buffy coat,…”	**Low**	**ACD**	**CaCl**_**2**_	**10% of 2.94+/-1.9 × 10**^**6**^	**2.94+/-1.9 × 10**^**6**^
Berger et al. (2019)	**Leukocyte level: mean 0.1 × 10**^**3**^**/μ L (SD 0.1)** (Supplementary material)	**Low**	**CPD**	**Freeze-and-thaw**	**20% of0.875 × 10**^**6**^**(Young group)20% of3.5 × 10**^**6**^**(Old group)**	**0.875 × 10**^**6**^
						**3.5 × 10**^**6**^
Wang et al. (2012)	**Leukocyte level:** 0.37 ± 0.14 ×10^9^/L Leukocyte concentration in PRP significantly lower than that in whole blood (*P* < 0.001). ( See article Table 2)	**Low**	**EDTA**	**Throm.+CaCl**_**2**_	**10% of 1.5–3.0 × 10**^**6**^	**2.25x 10**^**6**^
**Mean optimal PRP concentration**						**2.7 × 10**^**6**^

Interestingly, this review shows that the *Low Leukocyte Group* observed a substantially higher mean optimal PRP concentration for cell proliferation than the *High Leukocyte Group*, 2.7 ×10^6^, and 0.81 ×10^6^ plt/µL, respectively (Tables 5 and 6). However, there are substantial variations within each group. In *The Low Leukocyte Group,* Haynesworth et al. (2002) observed a relatively high optimal PRP concentration (10% of 1.6 ×10^6^) (Table 5). In *The Low Leukocyte Group*, Graziani et al. (2006) and Anitua, Cugat & Sánchez (2018) and Anitua et al. (2009) observed only 0.570 × 10 ^6^ and0.767 ± 95 × 10 ^6^plt/µL as the optimal concentration, respectively. It is worth pointing out that Graziani et al. (2006) and Anitua, Cugat & Sánchez (2018) and Anitua et al. (2009) deviate from the rest in this group, using a higher PRP-to-media ratio, namely 33.3% and 20% (Vol/Vol), respectively. A similar result is seen in Berger et al. (2010) in the “young group” (Table 6).

Generally, fibrinogen levels are not clarified in the selected studies. An assessment of the role of fibrinogen in PRP for proliferation in vitro is, therefore, not included in this review.

#### Anticoagulants and PRP activations

When it comes to anticoagulants, no tendencies or pattern are seen regarding optimal PRP concentration and proliferation. Different anticoagulant and activation methods are used in studies that both advocate lower and higher concentrations (Tables 5 and 6). ACD is the most widely used anticoagulant for the purpose; next are sodium citrate and GPD. Only one used EDTA.

The activation methods used are thrombin, calcium compounds (CaCl_2_ or Ca.glyconate), a combination of thrombin and Ca-compounds, or the freeze-and-thaw method. Only one study did not activate the PRP but applied the PRP directly to the cell culture (Tables 5 and 6). Jo et al. (2012) observed substantial higher proliferation rate when thrombin and calcium gluconate was combined compared to calcium gluconate alone. However, no clear tendencies or pattern is seen regarding different activation methods and the optimal PRP concentration (Tables 5 and 6).

### Variation due to blood donors (age and gender)

Berger et al. (2019) divided the blood donors in a “young group” (mean age 27 ±5) and an “old group” (mean age of 63 ±11) and observed an age-dependent optimal PRP concentration, 20% of 0.875 ×10^6^ and 3.5 x 10 ^*μ*^ plt/µL, respectively. These findings are comparable with Anitua et al. (2009) who used the same PRP volume (20%), cell type (fibroblasts), and blood donors characterized as “young” (Table 7). The six studies that involved blood donors with similar age as the “young group” in Berger et al. (2019) (approx. 25-35 y/o), had a mean optimal PRP concentration of 0.88 ×10^6^ plt/µL (Table 7, bold&italic). However, this age-dependence is weakly indicated in the included studies, because the studies that involved “young” donors applied different PRP-to-media ratio. Besides, Wang et al. (2019) observed optimal concentration in the range of 1.5–3.0 ×10^6^ plt/µL with a mean donor age of 39.2 ±5.8. Also Jo et al. (2012) observed a high optimal concentration with blood donors of 52.7 ±19.2-just slightly over the age limit (50y/o) of Berger et al. (2019) (Table 7).

**Table 7:** Age of blood donors and proliferative response.

**Study**	**Number ofblood donors**	**Age (y.)blood donor**	**Gender**	**Cell type**	**Optimal PRP concentrationcell proliferation(Vol/Vol and plt/ μ L or fold)**
Haynesworth et al. (2002)	No information	No information	No information	Fibroblasts	**10% of 1.6 × 10**^**6**^**(5 folds over baseline)**
Graziani et al. (2006)	3	***24-29***	Yes	Fibroblasts	**33.3% of 2.5x ≈ 0.570 × 10**^**6**^
Rughetti et al. (2008)	8	No information	No information	Endothelial cells	**1.25 × 10**^**6**^**(PRP/media ratio unclear)**
Anitua et al. (2009)	2	***“Young”***	No information	Fibroblasts (skin and synovium)	**20% of 0.767 ± 95 × 10**^**6**^**and0.404 ± 39 × 10**^**6**^
Hsu, Kuo & Tseng (2009)	20	***29.3 ± 6.5***	No information	Fibroblasts (PDL)	**5% of 1.124 × 10**^**6**^**of5% (Vol/Vol)**
Mishra et al. (2009)	No	No information	No information	Fibroblasts (skin)	**10% of 1 × 10**^**6**^
Jo et al. (2012)	9	52.7 ± 19.2	No information	Tenocytes	**10% of 4.00 × 10**^**6**^**(CaCl**_**2**_**)and10% of 8.00 × 10**^**6**^**(CaCl**_**2**_**+ thrombin)**
Mazzocca et al. (2012)	8	***31.6 ± 10.9***	No information	Tenocytes	**10% of PRP**_**LP**_**: 382.0+/-111.6 × 10**^**3**^**10% of PRP**_**DS**_**: 472.6+/-224.2 × 10**^**3**^
Chen et al. (2012)	10 Pooled	No information	No information	Dental pulp stem cells	**5% of 1.0 × 10**^**6**^**5% of 1.124 × 10**^**6**^**of5% (Vol/Vol)**
Wang et al. (2012)	3	***(24, 26, 41)Mean: 28.3***	No information	Tenocytes	**10% of 1.5-1.9 × 10**^**6**^**(4x)**
Sadoghi et al. (2013)	No	No information	No information	Unclear	**5-fold ≈ 1.25 × 10**^**6**^**(PRP/media ratio unclear)**
Amable et al. (2014)	3 pr. pool	No information	No information	HMSC	**10% of 1 × 10**^**6**^
Giusti et al. (2014)	3	(52, 30, 50) Mean: 44	M: 2 F: 1	Tenocytes	**0.5 × 10**^**6**^**(PRP/media ratio unclear)**
Tavassoli-Hojjati et al. (2016)	1	No information	No information	Fibroblasts (periodontal ligament)	**5% of 1.194 × 10**^**6**^
Berger et al. (2019)	14 Pooled	***27 ± 5 (Young group)***	M: 4, F: 2 M: 2, F: 6	Fibroblasts (Achilles, patellar, and palmaris)	**20% of0.875 × 10**^**6**^**(Young group)20% of3.5 × 10**^**6**^**(Old group)**
		63 ± 11 (Old group)			
Wang et al. (2012)	8 Individual	39.3 ± 5.8	M: 4 F: 4	HMSC (bone marrow)	**10% of 1.5 to 3.0 × 10**^**6**^

Possible differences due to gender cannot be assessed since most of the studies do not state gender.

## Discussion

### PRP-to-culture-media ratio: a probable bias

The author hypothesizes that the main course of this difference is due to variations in the PRP-to-culture-media ratio (Vol/Vol). As seen in *The Fixed PRP Concentration Group*, the highest PRP concentrations implied significant lower nutrition levels in the culture wells because of the diluting technique. This point is seen in Hsu, Kuo & Tseng (2009), Amable et al. (2014) and is particularly obvious in the study of Tavassoli-Hojjati et al. (2016). In the latter case, PRP (not lysate) corresponding to a platelet concentration of 1,194,000/µL was mixed with MDEM to process PRP concentrations of 0.1%, 5%, and 50% in which the cells were cultured. The optimal concentration of 5% contained 45% more nutrition than the PRP of 50%. A comparable pattern is seen in Giusti et al. (2014), who initially produced a PRP that contained 4.5 ×10^6^ to 6 ×10^6^ plt/µL. To make different concentrations of PRP-lysate, the initial PRP-lysate was diluted in DMEM + 1% FBS. Then, starved cells were exposed to (i.e., cultured in) the different PRP concentrations. Although a statistical assessment is not possible, the optimal PRP-lysate of 0.5 × 10 ^6^plt/µL had significantly more nutrition than the highest concentration (5 ×10^6^ plt/µL).

The comparable finding is revealed in *The Fixed PRP Volume Group*. The studies that advocate moderate platelet concentrations as most effective in inducing cell proliferation tended to have a higher fixed volume of PRP and, thus, a lower volume of culture media throughout the experiment. Graziani et al. (2006) used 100 µl media and 50 µl PRP of any concentrations, which mean a PRP part of 33.3% (Vol/Vol). The PRP-lysate containing 2.5× and 3.5× was diluted in extra DMEM to obtain the appropriate concentration. The highest concentration—PRP-max (4.2–5.5×)—received no extra MDEM. The cell cultures treated with lower platelet concentrations (2.5× and 3.5×) received substantially more nutrition than the group of PRP-max. This point is also seen in Anitua et al. (2009) and Berger et al. (2019) who kept the PRP-to-media ratio constant at 20% over 80% and observed a platelet concentration of 0.767 ± .95 and 0.875 (young group) × 10^6^ plt/µL as most effective—approximately half of what is described in Haynesworth et al. (2002), Rughetti et al. (2008) and Sadoghi et al. (2013). Compared to Haynesworth et al. (2002), Jo et al. (2012) and Wang et al. (2019) who utilized a 10/90 ratio (Vol/Vol), Graziani et al. (2006) and Anitua et al. (2009) used about 23% and 10% less nutrition, respectively.

### A tipping point at 10% PRP?

PRP volume over 10% might be critical in vitro. We have already seen that optimal platelet concentration in both Graziani et al. (2006) and Anitua et al. (2009) was significantly lower than in those who utilized a PRP concentration of 10% (Vol/Vol). Mishra et al. (2009) diluted PRP-lysates of 1 million plt/ µL to MDEM at concentrations of 0.1%, 1% 5%, 10%, and 20% (Vol/Vol) and observed the highest cell proliferation in the 10% PRP media. When 20% of PRP was used, they observed a significant decline in proliferation in the fibroblast culture. Comparable results were seen in Amable et al. (2014) and Haynesworth et al. (2002) when 20% PRP was used and Hsu, Kuo & Tseng (2009) when the PRP concentration was increased from 5% to 15%.However, (Chen et al., 2012) deviates from this prominent tendency and observed maximal proliferation when 5%, not 10%, PRP was applied culturing dental pulp stem cells.

These findings are of importance, taking into consideration the dramatic increase in nutrition requirement that happens when a cell undergoes growth and proliferation compared to its quiescent state (Palm & Thompson, 2017). A large quantity of nucleotides, amino acids, and lipids are required when a cell goes from the S_0_ state into the anabolic phases and proliferates into two viable daughter cells (Van der Heiden, Cantley & Thompson, 2009). Therefore, there is reason to speculate that the PRP-to-media ratio in the studies that advocated a low or moderate platelet concentration resulted in a lack of cellular nutrition and possible starvation.

### Cell density per well

The cell density per well (volume) may also be a bias factor, since an initial higher number of cells will require more nutrition for a prolonged proliferation and viability. Amable et al. (2014) and Tavassoli-Hojjati et al. (2016) utilized a 24-wells plate in their experiments, but seeded 1 ×10^3^ and 50 ×10^3^ cells/well, respectively. This may partly explain that the optimal platelet concentration according to Amable et al. (2014) is 5-6 times higher than according to Tavassoli-Hojjati et al. (2016) (10% of 2.94 ± −1.9 ×10^6^ plt/µL versus 5% of 1.194 ×10^6^ plt/µL, respectively). With the exception of Amable et al. (2014) and Graziani et al. (2006), the included studies give no exact information about the cell density per well. In general, information about type of culture plate (most often “96-wells plate”) and the number of cells/well or cells/cm^2^ is provided. However, the working volume inside each well is stretchable and allows for nutrition variation in each experiment. Therefore, the studies do not allow statistical analysis of this issue (Table 8).

**Table 8:** Cell density in culture. The values in al the studies are given in ×10^3^.

Haynesworth et al. (2002)	3 × 10^3^cells/cm^2^
Graziani et al. (2006)	1 ×10^3^ cells/well into 96-well plates 100ul DMEM + 50 PRP+DMEM/well
Rughetti et al. (2008)	1.5 ×10^3^ cells/well into 96-well plates
Anitua et al. (2009)	9.5 × 10^3^ cells/well into 24-well plates (Briefly)
Hsu, Kuo & Tseng (2009)	1 ×10^3^ cells/well
Mishra et al. (2009)	5 ×10^3^ cells/well into 96-wells plate.
Chen et al. (2012)	2 ×10^3^ cells/well into 96-well plates (normal medium) 5 ×10^3^ cells/well into 96-well plates cells/well (odonto/osteogenic differentiation medium)
Jo et al. (2012)	1 ×10^3^ cells/cm^2^
Mazzocca et al. (2012)	2,5 ×10^3^ cells/cm^2^
Wang et al. (2012)	4 × 10^3^cells/cm2 into 96-well plates
Sadoghi et al. (2013)	500 × 10^3^ cells into 6-well plates
Amable et al. (2014)	6 × 10^3^ cells/mL into 24-well plates
Giusti et al. (2014)	1 × 10^3^cells/well into a 96-well plate (Briefly)
Tavassoli-Hojjati et al. (2016)	50 × 10^3^ cells/well into five 24-well plates
Berger et al. (2019)	1 ×10^3^ cells/cm^2^ within collagen-coated, multi-well plates (Corning), using expansion medium
Wang et al. (2019)	10 ×10^3^/well into 96-well plates (five wells in each group)

### PRP processing method and biases

As often pointed out in the literature, there is no standardized PRP processing protocol. This heterogeneity in PRP protocol has led to a diversity of PRP products with different biological and biochemical characteristics. (Dohan Ehrenfest, Rasmusson & Albrektsson, 2009) have systematized different PRP products in four main categories: Pure platelet-rich plasma (P-PRP), Leukocyte- and platelet-rich plasma (L-PRP), Pure platelet-rich fibrin (P-PRF), and Leukocyte- and platelet-rich fibrin (L-PRF).

#### Spinning techniques as a source of bias

As previously said, the studies do not allow a systematic comparison when it comes to spinning force and time. A main focus regarding g-force and spinning time is to optimize the platelet yield (concentration) and to achieve high platelet viability. A protocol leading to a low or moderate PRP concentration may, in vitro, indirectly cause a bias later in the experiment: A too low or moderate final PRP concentration will force the researchers into the design of *The Fixed PRP Concentration Group* if higher concentrations should be tested—an experimental design that the author considers encumbered with biases. This seems to be the case in several of the studies in The Fixed PRP Concentration Group. Amable et al. (2014) and Giusti et al. (2014) are exceptions (Table 3). This is also the case in some of the studies within The Fixed PRP Volume Group when higher PRP concentration had to be obtained by increasing the PRP volume at the expense of the media volume (Haynesworth et al., 2002).

Regarding platelet viability, research shows that a too rough spin may activate the platelets during the process and lead to an early exocytosis of growth factors into the plasma, and thus undermine the regenerative effect of the PRP. A centrifuge spin lower that 3000 g is consider as a soft spin, and most of the studies seemed to utilize a spinning technique in the soft range. Two studies state a g-force higher than 3000 g (Anitua et al., 2009; Giusti et al., 2014).

Several studies have addressed the issue of the optimal spinning technique for high platelet yield, and there seem to be several paths to the goal (Amable et al., 2014; Araki et al., 2012; Perez et al., 2014). Because of deficient information about the spinning technique in several of the studies and the limitation of the paper, this subject will not be further treated.

### *Leukocytes* —*A deceitful biological component in vitro*

The use of autologous leukocyte enriched PRP (L-PRP) for cell proliferation  in vivo has both advocates and opponents (Brubaker et al., 2011; Dohan et al., 2006b; Hsu, Kuo & Tseng, 2009). In vitro, which generally is heterologous, the presence of leukocytes seems to represent a bias that prohibits proliferation at higher concentrations. The review demonstrates that The Low Leukocyte Group observed a substantially higher mean optimal PRP concentration for cell proliferation than The High Leukocyte Group. However, Graziani et al. (2007) observed a very low PRP concentration as optimal (0.570 ×10^6^ plt/µL), and Anitua et al. (2009) found no significant difference between PRP2x (0.404 ± 39 × 10^6^ plt/µL) and PRPmax (0.767 ± 95 × 10^6^ plt/µL). As mentioned previously, these research teams used a substantially higher PRP-to-media ratio compared to the rest of the Low Leukocyte Group, 33.3/66.6, and 20/80 (Vol/Vol), respectively. This seems to underline the importance of a low PRP-to-media ratio to avoid a lack of nutrition and, thus, best mimic the in vivo condition.

A possible explanation for the difference between The Low Leukocyte Group and The High Leukocyte Group is that degranulation of the leukocytes induces several substances that may prohibit proliferation and stimulate apoptosis—especially substances such as TNF-*α* and IL-1*β*, but also proteases and hydrolases, among others (Hsu, Kuo & Tseng, 2009; Jia et al., 2018). Also, Jo et al. (2012), with reference to Zimmermann et al. (2008), advocate leukocyte-reduced PRP products as most adequate when studying the leukocyte-independent effects of PRP. The upper leukocyte limit for in vitro studies is difficult to determine. Pillitteri et al. (2007) found that thrombin-induced IL-1 production could be detected at as low a level as 1 leukocyte per 5 ×10^5^ platelets, which indicates that leukocyte depletion is preferable. Unfortunately, the included studies do not allow assessing an exact upper limit of the leukocyte level for in vitro experiments.

#### Anticoagulants and activation method

Also, the choice of anticoagulant (Do Amaral et al., 2016), different activation methods (Berger et al., 2019; Jo et al., 2012; Tavassoli-Hojjati et al., 2016), pH condition (Mishra et al., 2009), have been thematized in the literature as co-elements that can increase or decrease the effect of PRP. This review does not illustrate any pattern or tendencies. Tables 5 and 6 show that different activation methods and anticoagulants have been applied in the studies that measured both a high and low optimal PRP concentration. However, this review does not exclude these aspects as possible factors.

### Experimental critics and further research

As shown, two main types of experimental technique are utilized: *The Fixed PRP Concentration Group* leads to severe variation in nutrition availability in the culture. On the other side, *The Fixed PRP Volume Group* kept the nutrition level relatively constant throughout the experiment by limiting the variation within a smaller ratio. The latter group should be considered as the most accurate for in vivo conditions. The exact number of cells per well and the volume of the well are factors that should be appreciated since an initial smaller number of cells/mL would require less nutrition to proliferate compared to a high number. Despite the possible benefits in vivo, the author considers leukocytes present in PRP as a bias in vitro—especially when the PRP is leukocyte enriched (L-PRP). To evaluate the leukocyte-independent effect of PRP in vitro, the leukocyte levels should be reduced, preferably depleted.

Based on the above discussion, the following basic experimental principles for further in vitro studies are recommended.

• The PRP/media ratio should be kept fixed throughout the experiment to minimize nutritional variations at different PRP concentrations.
• The PRP/media ratio should provide a sufficient nutrition supply to prevent cellular starvation, that is, PRP ≤ 10% (Vol/Vol). This implies that the initial PRP concentration should be high since an increase of concentration by increased volume is not recommended.
• The cell density (cells/mL) should be defined, that is, both the number of cells per well and nutrition volume should be clarified.
• Leukocyte level should be kept low, preferable depleted (<0.1 plt/µL).

### In vivo appraisal

In vitro studies should always be utilized with care. However, since the studies reviewed above aimed to point out or suggest an ideal platelet concentration in PRP treatment, the optimal platelet concentration in vivo should be discussed.

According to Van der Heiden, Cantley & Thompson (2009), the cells in multicellular organisms, including mammals, are normally exposed to a constant flow of nutrition supply. When PRP is injected in a tissue site and growth factors released, the arterial and capillary system will provide a constant flow of nutrition. The in vivo situation are, therefore, markedly different from the in vitro condition in which the nutrition supply is fixed and limited. In vivo, the growth factors will also gradually be diffused and transported out of the target area relatively quickly, which leads to a decline in levels of growth factors within hours, even if the molecular weight of the growth factors is high (6–150 kDa) (Kiritsy, Lynch & Lynch, 1993). Based on these perspectives, The fixed PRP Volume Group that uses 10% PRP volume at all concentrations, seems to best mimic the conditions in vivo.

If we pick out the five studies that combined low leukocyte levels with a fixed 10% PRP volume, the mean optimal PRP concentration for cell proliferation is 3.7 ×10^6^ plt/µL. The median is 2.94 ×10^6^ plt/µL (Table 9). Of course, no general or final conclusion can be drawn from these numbers regarding optimal PRP concentration in vivo. The table is statistically limited, and, as the review has shown, different cell types and tissue sites respond very differently regarding optimal PRP concentration. However, based on the above bias considerations, Table 9 might point against higher PRP levels than some of the “low” and “moderate” studies do.

**Table 9:** The mean optimal PRP concentration of selected studies. The selection of the studies is based on the two of the four criterias above: PRP volume ≤10% and low leukocyte levels.

**Study**	**Leucocyte characteristic**	**PRP to media ratio (Vol/Vol)Optimal concentration (plt/ μ L or fold)**	**Number used for calculation**
Jo et al. (2012)	**Low**	**10% of 4.00 × 10**^**6**^**(Ca. gluc.)and10% of 8.00 × 10**^**6**^**(Ca.gluc. + throm.)**	**4.00 × 10**^**6**^
			**8.00 × 10**^**6**^
Wang et al. (2012)	**Low (?)**	**10% of 1.5-1.9 × 10**^**6**^**(4x)**	**1.7 × 10**^**6**^
Amable et al. (2014)	**Low(?)**	**10% of 2.94+/-1.9 × 10**^**6**^	**2.94+/-1.9 × 10**^**6**^
Wang et al. (2019)	**Low**	**10% of 1.5-3.0 × 10**^**6**^	**2.25x 10**^**6**^
**Mean/median platelet concentration for cell proliferation**			**3.7/2.94 × 10**^**6**^

Still, extremely high platelet concentrations could be inadequate in injection sites where capillary density is low. Dernek et al. (2017) tested the effect of PRP clinically on knee osteoarthritis at 1 million plt/ µL (group 1) and 3 million plt/ µL (group 2). Regarding Western Ontario and McMaster University’s Osteoarthritis Index (WOMAC) scores, both groups improved significantly, but no significant difference was seen between the groups. Even if this study is based on a low volume of patients and is not randomized, it indicates that PRP of 1 million plt/ µL could be sufficient in intra-articular injections due to the low capillary density in these sites.

In vivo, on the other hand, the ideal leukocyte level is more complicated, and seems to be both cell-, tissue site-, and disease-dependent. Zhou et al. (2015) argues that L-PRP might be more beneficial for acute tendon injury, while P-PRP with a moderate leukocyte level is better suited in tendinopathic situations. However, this issue exceeding the limit of this paper.

Further research, cell type by cell type, tissue site by tissue site, in vitro and in vivo, is required to conclude on these questions.

### Limitations of this review

This review has several limitations that may pose a risk of possible biases:

Research in the last two decades strongly suggests that not only platelet concentration but also other biological qualities are crucial in regard to the effect of PRP in vivo (Dohan et al., 2006a). Especially the role of fibrinogen/fibrin (Dohan et al., 2006a; Dohan et al., 2006b) and number of monocytes (Dohan et al., 2006c) have been highlighted as important co-elements. This review has not incorporated these aspects since several studies have not metrically clarified other biological properties of the PRP than platelet concentration.

## Conclusions

The in vitro studies here reviewed states almost consistently that PRP stimulates the proliferation of the human cell. This observation is also the case regarding cell motility and exocytosis of several important regenerative extracellular ground substances, for example, collagen type I and III, HA, and so forth.

The studies diverge severely regarding the optimal PRP concentration for cell proliferation in vitro, due to different PRP production methods, PRP product characteristics, culture techniques and cell types and tissue sites. However, two main biases are pointed out in this review that might explain the detrimental effects at higher platelet concentrations in some of the studies:

### Bias regarding culturing technique

The review reveals two main types of culture techniques utilized: *The Fixed PRP Concentration Group* used a fixed PRP concentration and altered the platelet concentration by adding the different volumes of this PRP-lysate to the culture. *The Fixed PRP Volume Group* used a fixed PRP-to-media ratio (Vol/Vol) throughout the experiment and altered the PRP concentration in the PRP volume. An overall trait is seen: When the PRP concentration increases, the volume of culture media (nutrition) decreases, and lower optimal concentration for cell proliferation is observed. This is particularly prominent in group 1, *The Fixed PRP Concentration Group,* due to the diluting technique. We hypothesize that the techniques used by some studies led to adverse growth conditions and even cell starvation when high platelet concentrations were tested. Due to the constant nutrition supply and rapid diffusion of growth factors that normally occur in mammal tissue, the author considers the studies that used a fixed PRP-to-media ratio of 10%/90% (Vol/Vol) to best mimic the situation in vivo.

### Bias regarding The PRP processing method (leukocyte levels)

The review shows that researchers that used a protocol that provided PRP containing low levels of leukocytes observed a substantial higher optimal mean PRP concentration than teams in The High Leukocyte Group, possibly due to degranulation of substances in the leukocytes that prohibiting proliferation and promoting apoptosis.

These two biases may explain the bell-shaped effect of PRP with an optimal concentration of approx. 1 ×10^6^ plt/µL and the detrimental effects at higher platelet concentrations observed in several studies. The high PRP to media ratio (Vol/Vol) and/or high leukocyte levels may also explain the relatively low optimal PRP concentration observed in some studies, for example, Graziani et al. (2006) and Giusti et al. (2014).

No prominent tendencies were seen regarding the use of anticoagulant, activation method, and blood donor (age and sex) in the studies. However, these aspects should not be excluded.

If in vitro studies should be a wizard for developing PRP treatment in the future, the following basic experimental principles are recommended.

• The PRP/media ratio should be kept fixed throughout the experiment to minimize nutritional variations at different PRP concentrations.
• The PRP/media ratio should provide a sufficient nutrition supply to prevent cellular starvation, that is, PRP ≤ 10% (Vol/Vol). This implies an initial high PRP concentration since an increase of concentration by increased PRP volume is not recommended.
• The cell density (cells/mL) should be defined.
• Leukocyte level should be kept low, preferable depleted (<0.1 plt/µL).

An appraisal of the optimal PRP concentration in vivo is challenging, and the heterogeneity among the studies do not allow any strong suggestions. If we pick out the five studies that combined low leukocyte levels with a fixed 10% PRP volume (the two main biases), the mean optimal PRP concentration for cell proliferation is 3.7 ×10^6^ plt/µL. The median is 2.94 ×10^6^ plt/µL (Table 9). Of course, no general or final conclusion can be drawn from this number(s). The table is statistically limited and, as the review has showed, different cell types and tissue sites might respond different to PRP regarding optimal concentration. However, based on the above bias considerations and the abundant nutrition supply in vivo, this review indicates that PRP concentrations in the upper parts of the scale might be optimal or at least beneficial.

As mentioned in the introduction, several scientists advocate a moderate PRP concentration in vivo based on a few selected in vitro studies. The author does not recommend this approach and hope that this review will lead to a more critical and thorough interpretation of the in vitro studies.

Finally, it is questionable if one single ideal concentration exists. In the future, probably several optimal PRP concentrations will emerge, based on cell type, tissue site, perhaps age (Berger et al., 2019) and other factors.

##  Supplemental Information

10.7717/peerj.10303/supp-1 Supplemental Information 1 PRISMA 2009 flow diagram

10.7717/peerj.10303/supp-2 Supplemental Information 2 PRISMA 2009 checklist

## Additional Information and Declarations

### Data Availability

The following information was supplied regarding data availability: This is a literature review and does not contain raw data.
