# Supplementary material for: The optimal platelet concentration in platelet-rich plasma for proliferation of human cells in vitro—diversity, biases, and possible basic experimental principles for further research in the field: A review
Source: PeerJ. 2020 Nov 13;8:e10303. doi: 10.7717/peerj.10303 (PMC7668201; doi:10.7717/peerj.10303)
Supplement: Supplemental Information 1 [file peerj-08-10303-s001.pdf]

PRISMA 2009 Flow Diagram

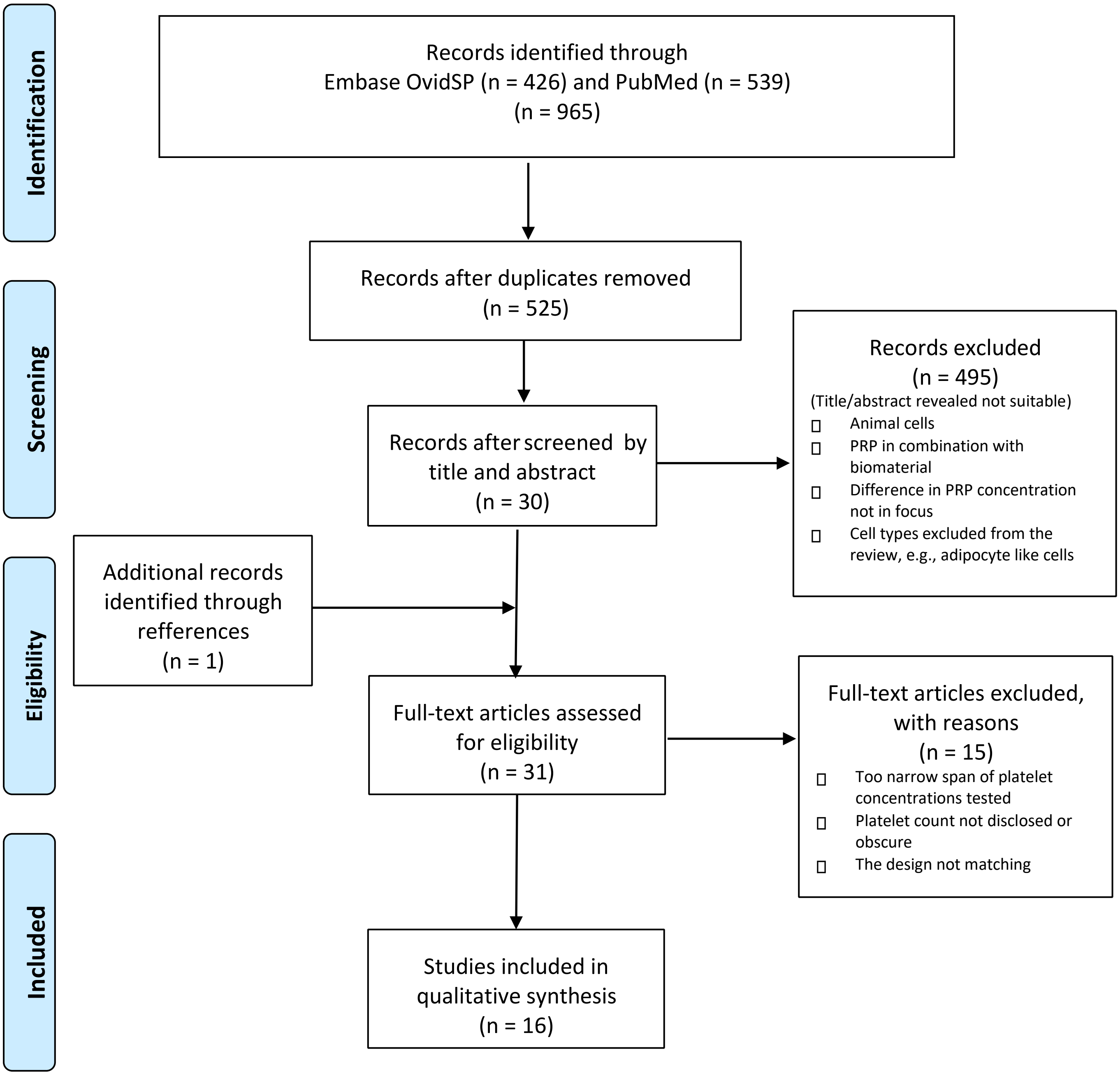

From: Moher D, Liberati A, Tetzlaff J, Altman DG, The PRISMA Group (2009). *P*referred *R*eporting *I*tems for *S*ystematic Reviews and *M*eta-

*A*nalyses: The PRISMA Statement. PLoS Med 6(7): e1000097. doi:10.1371/journal.pmed1000097

For more information, visit www.prisma-statement.org.
