# Supplementary material for: The optimal platelet concentration in platelet-rich plasma for proliferation of human cells in vitro—diversity, biases, and possible basic experimental principles for further research in the field: A review
Source: PeerJ. 2020 Nov 13;8:e10303. doi: 10.7717/peerj.10303 (PMC7668201; doi:10.7717/peerj.10303)
Supplement: Supplemental Information 2 [file peerj-08-10303-s002.pdf]

PRISMA 2009 Checklist

Section/topic	#	Checklist item	Reported on page #
**TITLE**			
Title	1	*Identify the report as a systematic review, meta-analysis, or both.* This is a literature review!	1
**ABSTRACT**			
Structured summary	2	*Provide a structured summary including, as applicable: background; objectives; data sources; study eligibility criteria, participants, and interventions; study appraisal and synthesis methods; results; limitations; conclusions and implications of key findings; systematic review registration number.*	1
**INTRODUCTION**			
Rationale	3	*Describe the rationale for the review in the context of what is already known.*	2
Objectives	4	*Provide an explicit statement of questions being addressed with reference to participants, interventions, comparisons, outcomes, and study design (PICOS).*	___
**METHODS**			
Protocol and registration	5	*Indicate if a review protocol exists, if and where it can be accessed (e.g., Web address), and, if available, provide registration information including registration number.*	____
Eligibility criteria	6	*Specify study characteristics (e.g., PICOS, length of follow-up) and report characteristics (e.g., years considered, language, publication status) used as criteria for eligibility, giving rationale.*	___
Information sources	7	*Describe all information sources (e.g., databases with dates of coverage, contact with study authors to identify additional studies) in the search and date last searched.*<br>A comprehensive search was carried out in EMBASE OvidSP and PubMed. Relevant articles found in references was also added. The search was performed in January 2020 and repeated in Mars 2020.	3
Search	8	Present full electronic search strategy for at least one database, including any limits used, such that it could be repeated.<br>The search terms used included: “platelet-rich plasma” OR “platelet concentrate*” OR “thrombocyte concentrate*” AND proliferation AND “in vitro”.	3
Study selection	9	*State the process for selecting studies (i.e., screening, eligibility, included in systematic review, and, if applicable, included in the meta-analysis).*<br>In total 965 records were identified: 426 studies in EMBASE OvidSP, 539 in Pubmed. The duplicates in the two bases were excluded, the initial number of studies was 525. After the first screening (title and abstract), 495 studies were excluded, frequently due to the use of animal cells, no focus on different PRP concentration, or because the design combined PRP with different types of biomaterials, etc. Additional 1 records were identified in references. The remaining 31 papers were included for full-text screening, of which 16 papers were included for the final analysis.	2
Data collection process	10	*Describe method of data extraction from reports (e.g., piloted forms, independently, in duplicate) and any processes for obtaining and confirming data from investigators.*	___
Data items	11	*List and define all variables for which data were sought (e.g., PICOS, funding sources) and any assumptions and simplifications made.*	____

Risk of bias in individual studies	12	*Describe methods used for assessing risk of bias of individual studies (including specification of whether this was done at the study or outcome level), and how this information is to be used in any data synthesis.*<br>The author applied as a starting point the principles of Good Cell Culture Practice (GCCP), and further developed these principles to the actual field. Following basic parameters was selected as potential areas of biases:<br>1. Number of wells used for testing of each PRP concentration<br>2. Duration of PRP exposure<br>3. Materials used according to the manufacturers' instructions<br>4. Sample size (number of blood donors)<br>5. Cell type(s) tested<br>6. Cell site origin<br>7. Cell number per well<br>8. PRP to media ratio	4, 9-10
Summary measures	13	*State the principal summary measures (e.g., risk ratio, difference in means).*	___
Synthesis of results	14	*Describe the methods of handling data and combining results of studies, if done, including measures of consistency (e.g., I^2) for each meta-analysis*.<br>Studies was tabulated and discussed chronological (publishing year) and alphabetically in each year group.	4

Section/topic	#	Checklist item	Reported on page #
Risk of bias across studies	15	*Specify any assessment of risk of bias that may affect the cumulative evidence (e.g., publication bias, selective reporting within studies).*	5
Additional analyses	16	*Describe methods of additional analyses (e.g., sensitivity or subgroup analyses, meta-regression), if done, indicating which were pre-specified.*	____
**RESULTS**			
Study selection	17	In total 965 records were identified: 426 studies in EMBASE OvidSP, 539 in PubMed. The duplicates in the two bases were excluded, the initial number of studies was 525. After the first screening (title and abstract), 495 studies were excluded, frequently due to the use of animal cells, no focus on different PRP concentration, or because the design combined PRP with different types of biomaterials, etc. Additional 2 records were identified in references. The remaining 30 papers were included for full-text screening, of which 16 papers were included for the final analysis.	3-6
3-6Study characteristics	18	Initial platelet concentration in PRP, Acivation method, different PRP concentration tested, time of PRP exposure, maximum proliferation	3-6

PRISMA 2009 Checklist

Risk of bias within studies	19	Generally, the 16 studies appeared relatively sound. Based on the bias assessment criteria mentioned above, 13 of the studies was classified as "low risk of biases", and 3 was classified as "moderate risk of biases". No studies were classified as "high risk of biases".	4-5
Results of individual studies	20	For all outcomes considered (benefits or harms), present, for each study: (a) simple summary data for each intervention group (b) effect estimates and confidence intervals, ideally with a forest plot.	5-10
Synthesis of results	21	*Present results of each meta-analysis done, including confidence intervals and measures of consistency.*	-
Risk of bias across studies	22	*Present results of any assessment of risk of bias across studies (see Item 15).*	10-12
Additional analysis	23	*Give results of additional analyses, if done (e.g., sensitivity or subgroup analyses, meta-regression [see Item 16]).*	——
**DISCUSSION**			
Summary of evidence	24	*Summarize the main findings including the strength of evidence for each main outcome; consider their relevance to key groups (e.g., healthcare providers, users, and policy makers).*	10-13
Limitations	25	*Discuss limitations at study and outcome level (e.g., risk of bias), and at review-level (e.g., incomplete retrieval of identified research, reporting bias).*	14
Conclusions	26	*Provide a general interpretation of the results in the context of other evidence, and implications for future research.*	14
**FUNDING**			
Funding	27	*Describe sources of funding for the systematic review and other support (e.g., supply of data); role of funders for the systematic review.*	15

From: Moher D, Liberati A, Tetzlaff J, Altman DG, The PRISMA Group (2009). Preferred Reporting Items for Systematic Reviews and Meta-Analyses: The PRISMA Statement. PLoS Med 6(7): e1000097. doi:10.1371/journal.pmed1000097

For more information, visit: **www.prisma-statement.org**.
